# Dynamical Characteristics of Isolated Donors, Acceptors, and Complex Defect Centers in Novel ZnO

**DOI:** 10.3390/nano15100749

**Published:** 2025-05-16

**Authors:** Devki N. Talwar, Piotr Becla

**Affiliations:** 1 Department of Physics, University of North Florida, 1 UNF Drive, Jacksonville, FL 32224, USA; 2 Department of Physics, Indiana University of Pennsylvania, 975 Oakland Avenue, 56 Weyandt Hall, Indiana, PA 15705, USA; 3 Department of Materials Science and Engineering, Massachusetts Institute of Technology, Cambridge, MA 02139, USA; becla@mit.edu

**Keywords:** site selectivity of defects in novel ZnO, Li and Al pair defects, Raman scattering, Fourier transform infrared spectroscopy, Green’s function, lattice dynamics

## Abstract

Novel wide-bandgap ZnO, BeO, and ZnBeO materials have recently gained considerable interest due to their stellar optoelectronic properties. These semiconductors are being used in developing high-resolution, flexible, transparent nanoelectronics/photonics and achieving high-power radio frequency modules for sensors/biosensors, photodetectors/solar cells, and resistive random-access memory applications. Despite earlier evidence of attaining p-type wz ZnO with N doping, the problem persists in achieving reproducible p-type conductivity. This issue is linked to charging compensation by intrinsic donors and/or background impurities. In ZnO: Al (Li), the vibrational features by infrared and Raman spectroscopy have been ascribed to the presence of isolated ${Al}_{Zn}{(Li}_{Zn})$ defects, nearest-neighbor (NN) ${[Al}_{Zn}{-N}_{O}$] pairs, and second NN ${[Al}_{Zn}{-O-Li}_{Zn};V_{Zn}-O{-Li}_{Zn}]$ complexes. However, no firm identification has been established. By integrating accurate perturbation models in a realistic Green’s function method, we have meticulously simulated the impurity vibrational modes of ${Al}_{Zn}$ ${(Li}_{Zn})$ and their bonding to form complexes with dopants as well as intrinsic defects. We strongly feel that these phonon features in doped ZnO will encourage spectroscopists to perform similar measurements to check our theoretical conjectures.

## 1. Introduction

High-performance electronic devices are essential to the modern world. There is almost no facet of our society that could not benefit from access to better quality and cheaper materials. In the selection process for device applications, scientists and engineers have always preferred those ingredients in a compound that are earth-abundant with low toxicity [1,2,3,4,5,6,7,8,9,10,11,12,13,14,15,16,17]. Oxygen (O) is an important member of the group of VI^A^ elements in the periodic table. It can form wide-bandgap (WBG) oxides with different constituents of the group II^B^ atoms (Be, Mg, Zn, and Cd). Novel composites of ${II}^{B}-{VI}^{A}$ are often classified as II-oxides (or II-Os). At ambient conditions, most II-Os are highly stable in the wurtzite (wz) or α-phase. They can be transformed into a rock salt (rs) structure under high pressure and to a metastable zinc blende (zb) or β-phase if grown on Si or GaAs (001) and (111) substrates [16].

BeO, MgO, ZnO, and CdO exhibit many characteristics identical to BN, AlN, GaN, and InN [1,2,3,4,5,6,7,8,9,10,11,12,13,14,15,16,17]. Similar to BN, BeO is an exceptionally hard material [18] and reveals unique phonon characteristics different from other II-Os. As compared to GaN and SiC, ZnO (with bandgap $E_{g}$ ~3.3 eV) has a few advantages due to a higher ~60 meV exciton-binding energy and availability as a large-size single crystal [15,16,17]. Higher cohesive energy, as well as a high melting point with strong bonding, implies that the degradation of ZnO-based electronic devices might not be an issue for high-temperature T operations. Another niche application accomplished recently is the fabrication of transparent thin-film transistors. Based on transparent conductive oxide (TCO) [19,20,21,22], these transistors are insensitive to visible light, where the protective covering to prevent light exposure can be eliminated. TCO has now emerged as one of the most promising contenders to indium tin oxide (ITO), owing to its vital optoelectronic properties with many benefits, including non-toxicity, availability of abundant raw material, and cost-effectiveness. A few issues in the development of TCO-based devices include difficulty in achieving reproducible p-type doping. Many reports suggest that the optical properties of ZnO are significantly influenced by the presence of intrinsic defects [23,24,25,26,27,28,29,30,31,32,33,34,35,36], including oxygen vacancy, zinc vacancy $(V_{O}$, $V_{Zn}),$ zinc interstitials $\left({Zn}_{i}\right),$ etc. It is academically and technologically important to study the roles of such defects for the development of reliable p-type doping.

In ZnO, group III (B, Al, Ga, and In) dopants have been used as donors to investigate electronic and vibrational characteristics [23,24]. In II–VI semiconductors, group I (Li, Cu, Ag) and group V (X = N, P, As, Sb) atoms are frequently exploited to make p-type materials. Earlier evidence of N-doped ZnO grown by MBE [16,25,26] paved the way for creating p–n junctions. However, the replication to achieve p-type ZnO has been and still is a challenge [16]. The experimental interpretation of the electronic and vibrational properties for ${Li}_{Zn},X_{O}$ as acceptors and ${Al}_{Zn}$ as donors have varied significantly [25,26]. Except for N, the mass and ionic radii of P, As, and Sb are much larger than the O atom [27]. This means that the vibrational modes of isolated $X_{O}$ will fall in the phonon frequency region of the perfect ZnO. Such vibrational features cannot be observed or analyzed accurately. Interestingly, the ZnO: P samples prepared by different techniques have exhibited the lowest resistivity [16]. The hole concentration and mobilities are usually small due to high activation energies and self-compensation by native impurities. Such intrinsic defects are suggested to create electrically inactive but stable complexes. In simulating the electrical properties of ZnO: X materials, many researchers [37] have considered anti-site $X_{Zn}$ defects, as they reduce the lattice strain caused by a mismatch between the ionic radii of X and O. Several reports also indicated that the size difference between X and O initiates deep acceptor states. The difficulty of achieving p-type ZnO: X is caused by the charge compensation by intrinsic donors and/or background impurities [37]. In ZnO: Al (Li), besides substitutional Al (${Al}_{Zn}$) (Li (${Li}_{Zn}$)) defects, the existence of many intrinsic impurities (viz.,$V_{O}$, ${Zn}_{i}$, $V_{Zn}$, $O_{i}$ and anti-site $O_{Zn}{,Zn}_{O}$) are proposed [24,25,26,27,28,29,30,31,32,33,34,35,36,37]. Force constants describing an interaction between an isolated ${Al}_{Zn}\;{(Li}_{Zn})\;$ defect and its nearest-neighbor (NN) host atoms are considerably higher (lower) than the perfect crystal. Moreover, the involvement of intrinsic impurities is suggested to form different complexes [24,25,26,27,28,29,30,31,32,33,34,35,36,37] with ${Al}_{Zn}\;{(Li}_{Zn})$. The observed vibrational features by Raman scattering and Fourier transform infrared (FTIR) spectroscopy have been attributed to NN ${(Al}_{Zn}{-N}_{O}$) and/or second NN ${(Al}_{Zn}{-O-V}_{Zn};{Al}_{Zn}{-O-Li}_{Zn})$ defect centers. However, no evidence exists for the involvement of intrinsic impurities in complex centers. Comprehending the role of trivalent B, Al, and monovalent Li dopants in ZnO is crucial. Appraising the association of ${Al}_{Zn},\;{{Bl}_{Zn}(Li}_{Zn})$ with intrinsic defects could play an important role in making n- and p-type materials with structural order and the required physical properties.

Different epitaxial growth methods have been employed in recent years to prepare ultrathin Zn_x_Be_1−x_O films [38,39,40,41,42,43,44,45,46,47]. The techniques that have been used included metal-organic chemical vapor deposition (MOCVD) [38,39,40], laser ablation [41], CVD, molecular beam epitaxy (MBE), plasma-assisted PA-MBE [42,43,44,45,46,47], etc. Many characterization efforts are also made for analyzing and monitoring their basic properties [48,49,50,51,52,53,54,55,56,57,58,59,60,61,62,63,64,65,66,67,68,69]. Several measurements have been performed by reflection high-energy electron diffraction (RHEED) [48], Auger electron spectroscopy (AES) [50,51,52], He^+^ Rutherford backscattering spectrometry (RBS) [53,54,55,56,57,58], atomic force microscopy (AFM) [59,60], high-resolution X-ray diffraction [61,62,63,64] (HR-XRD), transmission electron microscopy (XTEM) [65], photoluminescence (PL) [66,67], absorption, FTIR [60], Raman scattering spectroscopy (RSS) [65,66,67], spectroscopic ellipsometry (SE) [68,69], etc. These experimental methods have not only validated the crystal structures but also helped to evaluate the epifilm’s thickness, strain, electrical, and optical traits of intrinsic defects. Assimilation of ZnBeO epifilms in MQW and SLs has played an important role in the development of various electronic devices, including insulated gate bipolar transistors (IGBTs), transparent conductive films for efficient charge carrier transport, ultraviolet (UV) light-emitting diodes (LEDs), high-electron-mobility transistors (HEMTs), heterostructure field-effect transistors, optoelectronic gas sensors [1,2,3,4,5,6,7,8,9,10,11,12,13,14,15,16,17], etc. Integrating such devices into flexible transparent nanoelectronics/photonics has helped achieve high-resolution medical imaging tools for physicians to diagnose, treat, and rehabilitate illnesses and diseases. Many optoelectronic units are also being incorporated for creating high-power radio frequency modules, sensors/biosensors, photodetectors/solar cells, resistive random-access memory, and surface acoustic wave devices.

To study the lattice dynamics of perfect/imperfect semiconductors, two theoretical approaches are commonly adopted: (i) the microscopic or first principles methods [70,71,72,73,74,75,76,77,78,79], which start with an ionic potential screened by electron gas for deriving the structural and vibrational properties, and (ii) the macroscopic techniques that employ phenomenological models [80,81,82,83,84,85,86,87] in terms of general interatomic forces. Very few calculations using the density functional method (DFT) are known for the lattice dynamics of perfect II-Os [77,78,79]. Absolutely no studies exist on the vibrational properties of isolated and complex defect centers in ZnO. To comprehend the dynamical properties of impurities, many researchers have preferred using macroscopic methods in the framework of Green’s function (GF) formalism [80,81,82,83,84,85,86,87]. Careful analysis of local vibrational modes (LVMs) for donors and acceptors in semiconductors has revealed an important revelation. Changes in the NN impurity–host interactions can be ascribed to the electric field created (cf. Section 3) by charged impurities [84,85,86,87]. This important fact is used here for studying the LVMs of a ${Li}_{Zn}$ acceptor, $B_{Zn}\;and\;\;{Al}_{Zn}$ donors, and donor–acceptor pairs. The simulation of impurity vibrational modes by the GF method requires accurate phonon dispersions of perfect materials. We have adopted a rigid-ion model (RIM) [88] to calculate the phonon dispersions of zb ZnO and BeO. Eigen values and eigenvectors of perfect materials are carefully integrated to obtain the GF matrix elements (${\overset{⃡}{G}}^{o}$). Appropriate perturbation matrices ($\overset{⃡}{P}$) of isolated and complex defect centers are considered to study the impurity vibrational modes. Comparison of the theoretical results [81,82,83] with existing Raman scattering and/or FTIR data has helped to identify the nature of different types of defects in zb ZnO.

This paper aims to present the systematic results of GF calculations for the dynamical characteristics of various defects in ZnO. Different crystal structures are described in Section 2.1, with the possible existence of intrinsic defects in II-Os. For the lattice dynamics of perfect crystals, a realistic RIM [88] (cf. Section 2.2) is adopted, which includes both the short-range and long-range Coulomb interactions. Essential features of RIM are succinctly outlined in Section 2.3, Section 2.3.1, Section 2.3.2 and Section 2.3.3 for evaluating the ${\overset{⃡}{G}}^{o}$ and $\overset{⃡}{P}$ matrices [81,82,83]. The GF approach leads to Dyson’s equation to help examine the impurity-induced vibrational modes. For ZnO, the calculated results of the phonon dispersions $\omega_{j}(\vec{q})$ and density of states g(ω) are compared with the experimental data in Section 3.1. In defining $\overset{⃡}{P}$, we did not include changes in the Coulomb forces, as their long-range interactions would render the GF approach intractable. The perturbation matrices include only those changes in atomic masses at the impurity sites as well as the NN force constants. Group-theoretical arguments are used to block-diagonalize the $\overset{⃡}{P}$ and ${\overset{⃡}{G}}^{o}$(cf. Section 3) matrices for (i) single isolated defects of $T_{d}$ symmetry, (ii) NN pair defects of $C_{3v}$ symmetry, and (iii) second NN (NNN) complex defect centers of $C_{s}$ or $C_{2v}$ symmetry. A modified random element-iso-displacement (MREI) [89,90] model (see Section 3.2) is employed to describe the x-dependent two-phonon mode behavior of Zn_x_Be_1−x_O alloys. Impurity modes are evaluated in Zn_x_Be_1−x_O by the GFmethod at the extreme composition limits (i.e., x → 1 i.e., ZnO:Be ${Be}_{Zn}\sim \;775\;{\;cm}^{-1}\;$ and x → 0 for BeO:Zn ${Zn}_{Be}\sim \;280\;{\;cm}^{-1}).$ For the closest mass isoelectronic ${Be}_{Zn}\left(i\right)defect,$ we used the force variation correlation ∆t to obtain the impurity modes of the ${Li}_{Zn}\left(a^{-}\right)$ acceptor (cf. Section 3.2) and $B_{Zn}\left(d^{+}\right)\;donor$ in ZnO. The GF method has predicted LVMs of $B_{Zn}\left(d^{+}\right)$ or ${Al}_{Zn}\left(d^{+}\right)$ donors forming NN pairs with group V (X = N, P, As, Sb) acceptors. Similarly ${Li}_{Zn}\left(a^{-}\right)$ can be used as prospective dopants for creating NNN complexes with $B_{Zn}\left(d^{+}\right)$, ${Al}_{Zn}\left(d^{+}\right)$. Our results of LVMs for different impurity centers are valuable and encourage spectroscopists to check our theoretical conjectures. The concluding remarks are presented in Section 4 on the microstructures involving group III atoms as donors and group I or V as acceptors.

## 2. Theoretical Background

### 2.1. Crystal Structure

**Figure 1:**
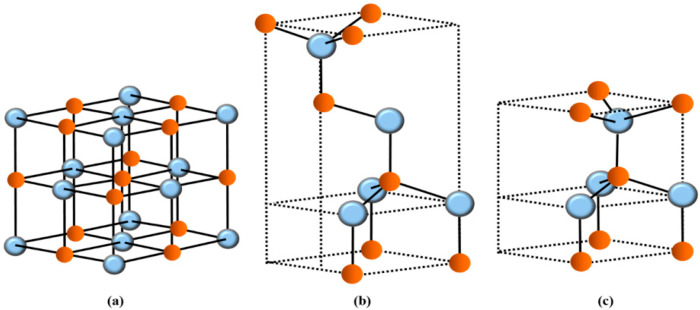
The crystal structures of ZnO and/or BeO materials: Rock salt (**a**), zinc blende (**b**), and wurtzite (**c**). The red-colored spheres represent the O atoms, while the sky blue-colored spheres represent either the Zn or Be atoms.

At high pressure, the ZnO (BeO) material usually occurs in the NaCl-like rs crystal structure with a space group $Fm\bar{3}m(O_{h}^{5})$ or in a B1 polymorph (see Figure 1a)). An ultrathin ZnO (BeO) film can exhibit the zb cubic phase if prepared on GaAs (001) and (111) or Si substrate [16]. The film has a mixture of tetrahedral covalent and ionic bonding with a space group of $F\bar{4}3m\left(T_{d}^{5}\right)$ or a B3 structure (see Figure 1b)). At ambient conditions of T and pressure P, the epitaxially grown ZnO (BeO) semiconductor material on most substrates typically occurs in a stable wz or B4 structure (see Figure 1c)) with space group $P6_{3}mc(C_{6v}^{4})$. The NN $T_{d}$ environment in both the wz and zb materials is very similar.

#### Intrinsic Defects in ZnO

ZnO is a prototypical n-type semiconductor that exhibits several fascinating physical and chemical properties. While the material is considered valuable for a variety of applications, the realization of many devices has been hindered, however, due to difficulties in achieving reliable and reproducible p-type ZnO. This complexity arises from the strong compensation caused by intrinsic $V_{O}$,$\;{Zn}_{i}$ $O_{Zn}$ donors, and/or background impurities. The n-type conductivity of $V_{O}$ is still being debated. The O-deficient ZnO has always behaved like an n-type without intentional doping. Theoretical studies [70,71,72,73,74,75,76,77,78,79] have indicated $V_{O}$ exhibiting the lowest formation enthalpy. Similar to many deep donors, $V_{O}$ in ZnO leads to a deep electronic state. At room temperature, $V_{O}\;$ cannot be ionized to contribute to n-type conductivity. Thus, the defect energetics of $V_{O}\;$ have created huge controversy [16]. We will address some of these issues by simulating the dynamical characteristics of various defect centers involving double dopants (e.g., acceptor atoms from Group I (Li) and donors from Group III (B, Al)) using GF methodology in the framework of a realistic RIM.

### 2.2. The Rigid-Ion Model

To treat the lattice dynamics of zb materials in the harmonic approximation, the invariance of potential energy with respect to rigid-body translations, rotations, and symmetry operations requires a minimum of two (A, B) NN force constants [91]. For GaAs, this two-parameter force constant model has failed, however, to reproduce the inelastic neutron scattering (INS) results [92] of $\omega_{j}(\vec{q})$. This prompted improvement in the oversimplified model by including interactions beyond the NNs. The upgraded RIM, proposed by Kunc [88], has accurately explained the INS data of $\omega_{j}(\vec{q})$ for GaAs [92].

The quantities of interest in RIM are the force constants ${\overset{⃡}{\Phi }}^{sC}[\equiv {\overset{⃡}{\Phi }}^{s}+{\overset{⃡}{\Phi }}^{C}]$ or dynamical ${\overset{⃡}{D}}^{sC}[\equiv {\overset{⃡}{D}}^{s}+{\overset{⃡}{D}}^{C}]$ matrices [88]. For zb materials of $T_{d}$ symmetry, the RIM includes short-range interactions (${\overset{⃡}{\Phi }}^{s}$) up to the second NN with ten parameters (A, B, $C_{\kappa }$, $D_{\kappa }$, $E_{\kappa }$ and $F_{\kappa }$; κ = 1, 2). A long-range Coulomb interaction (${\overset{⃡}{\Phi }}^{C})$ is also incorporated using an effective charge parameter $Z_{eff}\;(\equiv Z_{\kappa }e)$. In the harmonic approximation, the lattice vibrations $\omega_{j}(\vec{q})$ are obtained by solving the following equations of motion [88]:

(1) $$\omega_{j}^{2}(\vec{q})e_{\alpha }(\kappa |\vec{q}j)=\sum_{\kappa^{\prime }\beta }D_{\alpha \beta }^{sC}(\kappa \kappa^{\prime }|\vec{q})e_{\beta }(\kappa^{\prime }|\vec{q}j);\kappa ,\kappa^{\prime }=1,2,$$

where $D_{\alpha \beta }^{sC}(\kappa \kappa^{\prime }|\vec{q})\left[\equiv D_{\alpha \beta }^{s}(\kappa \kappa^{\prime }|\vec{q})+D_{\alpha \beta }^{C}(\kappa \kappa^{\prime }|\vec{q})\right]$ represents the dynamical matrix comprising of short- $D_{\alpha \beta }^{s}(\kappa \kappa^{\prime }|\vec{q})$ and long-range Coulomb $D_{\alpha \beta }^{s}(\kappa \kappa^{\prime }|\vec{q})$ interactions. For each mode of frequency $\omega_{j}\left(\vec{q}\right),$ the components of eigenvectors $e_{\alpha }(\kappa |\vec{q}j)$ in Equation (1) satisfy the familiar orthogonality [88]

(2a) $$\sum_{\alpha \kappa }e_{\alpha }^{*}(\kappa |\vec{q}j)e_{\alpha }(\kappa |{\vec{q}j}^{\prime })=\delta_{jj^{\prime }},$$

and closure relations

(2b) $$\sum_{j}e_{\alpha }^{*}(\kappa^{\prime }|\vec{q}j)e_{\beta }(\kappa |{\vec{q}j}^{\prime })=\delta_{{\kappa \kappa }^{\prime }}\delta_{\alpha \beta }.$$

Once the interatomic force constants (IFCs) $[A,B,C_{\kappa },D_{\kappa },E_{\kappa },F_{\kappa }$, and $Z_{eff}(\equiv Z_{\kappa }$e)] are evaluated for the zb ZnO [66] and BeO, it is straightforward to simulate $\omega_{j}(\vec{q})$ by using Equation (1).

### 2.3. Green’s Function Approach

As stated earlier, the impurity vibrational modes’ calculations by ab initio methods require heavy computation for isoelectronic Cd*Te*:O [75] defects, and it is much more cumbersome for non-isoelectronic (charged) impurities. One must note that in CdTe, no efforts are made by DFT for extracting an impurity–host interaction for ${Al}_{Cd}$ (d^+^) donors and ${Li}_{Cd}$ (a^−^) acceptors. In the GF methodology, however, by considering appropriate symmetries of different [81,82,83,84] defect centers, it is possible to visualize and identify those impurity modes that are optically active and remain localized around the defects. Detailed accounts of the dynamical properties for perfect/imperfect crystals by the GF method have been reported [81,82,83,84] elsewhere. Thus, our discussion on treating the impurity vibrational modes in zb ZnO, will be very brief and only for the purpose of establishing important notations to be used throughout this paper.

#### 2.3.1. The Perfect Lattice Green’s Functions

Using RIM [88], the perfect lattice GF ${\overset{⃡}{G}}^{o}$ of zb ZnO and/or BeO can be expressed in the matrix notation as:

(3) $$(\overset{⃡}{M}\omega^{2}-{\overset{⃡}{\Phi }}^{sC})\;{\overset{⃡}{G}}^{o}=\overset{⃡}{I},$$

where the eigenfrequencies of the host crystal are obtained by solving the equation:

(4) $$\det\;[(\overset{⃡}{I}\omega^{2}-{\overset{⃡}{D}}^{sC})]=\det{\left[{\overset{⃡}{G}}^{o}\left(\omega \right)\right]}^{-1}/\det\left[\overset{⃡}{M}\right].$$

The component form of the ${\overset{⃡}{G}}^{o}$ matrix is defined as:

(5) $$<l\kappa \left|G_{\alpha \beta }^{o}\left(\omega \right)\right|l^{i}\kappa^{\prime }>=\frac{1}{ℵ{\left(M_{\kappa }M_{\kappa^{\prime }}\right)}^{1/2}}\sum_{\vec{q}j}\frac{e_{\alpha }(\kappa |\vec{q}j)e_{\beta }^{*}(\kappa^{\prime }|\vec{q}j)}{{(\omega +i0}^{+}{)}^{2}-\omega_{j}^{2}(\vec{q})}\times exp\left\{i\vec{q}[\vec{x}\left(l\kappa \right)-\vec{x}\left(l^{\prime }\kappa^{\prime }\right)]\right\}\;,$$

where ℵ denotes the number of wave vectors, and $\vec{x}\left(l\kappa \right)$ is the equilibrium position vector of an atom (lκ). An infinitesimal positive imaginary value to ω is added for producing the retarded GF with a sinusoidal time dependence. The elements of ${\overset{⃡}{G}}^{o}$ (Equation (5)) can be expressed in terms of its real and imaginary parts [83]. The real part of the matrix $<l\kappa \left|{ReG}_{\alpha \beta }^{o}\left(\omega \right)\right|l^{i}\kappa^{\prime }>$ is the principal segment of Equation (5), while the imaginary part $<l\kappa \left|{ImG}_{\alpha \beta }^{o}\left(\omega \right)\right|l^{i}\kappa^{\prime }>$ can be expressed as:

(6) $$<l\kappa \left|{ImG}_{\alpha \beta }^{o}\left(\omega \right)\right|l^{i}\kappa^{\prime }>\frac{\pi }{ℵ{\left(M_{\kappa }M_{\kappa^{\prime }}\right)}^{\frac{1}{2}}}\sum_{\vec{q}j}e_{\alpha }\left(\kappa |\vec{q}j\right)e_{\beta }^{*}\left(\kappa^{\prime }|\vec{q}j\right)\;\times exp\left\{i\vec{q}[\vec{x}\left(l\kappa \right)-\vec{x}\left(l^{\prime }\kappa^{\prime }\right)]\right\}\times \delta \left(\omega^{2}-\omega_{j}^{2}\left(\vec{q}\right)\right).$$

Clearly, Equation (6) becomes zero outside the range of allowed phonon frequencies of the host crystal lattice. For the numerical calculations of GFs, we have followed the standard procedures by first obtaining the imaginary part from a sample of wave vectors $\vec{q}$ in the reduced BZ and then determining the real part via the links provided by Kramers–Krönig relations [81,82,83,84].

#### 2.3.2. The Imperfect Lattice Green’s Functions

Similar to the ${\overset{⃡}{G}}^{o}\;$ (cf. Section 2.3.1) of the perfect lattice, one can write the GF matrix elements for the imperfect crystal $\overset{⃡}{G}$ by using:

(7) $$[(\overset{⃡}{M}+∆\overset{⃡}{M})\omega^{2}-({\overset{⃡}{\Phi }}^{sC}+∆{\overset{⃡}{\Phi }}^{sC})]\;\overset{⃡}{G}=\overset{⃡}{I},$$

or equivalently in the form of a Dyson’s equation

(8) $$\overset{⃡}{G}\left(\omega \right)=[\overset{⃡}{I}-{\overset{⃡}{G}}^{o}(\omega )\;\overset{⃡}{P}(\omega ){]}^{-1}{\overset{⃡}{G}}^{o}\left(\omega \right).$$

In Equation (8), the term $\overset{⃡}{P}\left(\omega \right)[\equiv -∆\overset{⃡}{M}\omega^{2}+∆{\overset{⃡}{\Phi }}^{sC}]$ is the perturbation matrix caused by the defects. The quantities $∆\overset{⃡}{M}$ and $∆{\overset{⃡}{\Phi }}^{sC}[\equiv ∆{\overset{⃡}{\Phi }}^{s}+∆{\overset{⃡}{\Phi }}^{C}]$ represent the mass and force constant change matrices. Since the variations in the Coulomb interactions are set to zero $∆{\overset{⃡}{\Phi }}^{C}\;$= 0, we will consider only the mass change at the impurity sites and the variations in the NN impurity–host interactions (cf. Section 2.3.3) in defining $\overset{⃡}{P}$ for different defect centers. The impurity vibrational modes can be obtained by solving the equation [81,82,83,84]:

(9) $$det|[\overset{⃡}{I}-{\overset{⃡}{G}}^{o}(\omega )\;\overset{⃡}{P}\left(\omega \right)]|=0.$$

Equation (9) may provide the poles of $\overset{⃡}{G}\left(\omega \right)$ as follows: (i) as a LVM at an energy above the maximum phonon frequency of the bulk material, (ii) as a gap mode (GM) in the space between the acoustic and optic modes, and (iii) as an in-band mode falling within the host lattice phonons [60,61,62,63,64,65,66,67]. To simulate the impurity modes of various defect centers, we took advantage (cf. Section 2.3.3) of the symmetry-adapted algorithm [80,81]. This method has helped us compare theoretical results with spectroscopic data.

#### 2.3.3. Perturbation Matrices

**Figure 2:**
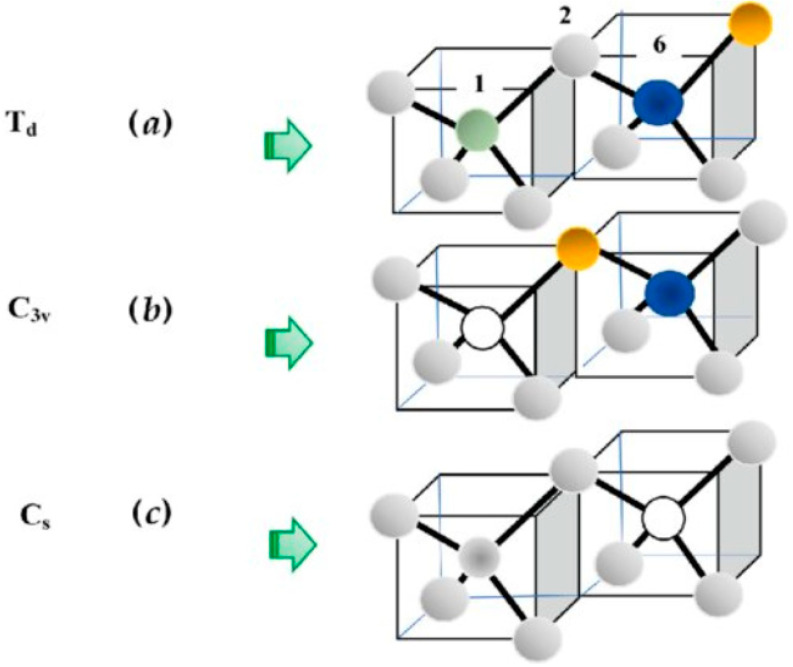
Perturbation models for (**a**) an isolated ($T_{d}$ symmetry) defect: The impurity atoms are occupying either the Zn atom on a site (κ = 1) with neighboring host O atoms on sites 2, 3, 4, 5; or the O atom on a site (κ = 2) with neighboring host Zn atoms on sites 1, 6, 7, 8 in ZnO. (**b**) A nearest-neighbor pair defect ($C_{3v}$ symmetry), where the two impurity atoms are occupying sites κ = 1 (Zn) and 2 (O), respectively. Impurity on the Zn site has host O atoms at sites 3, 4, and 5, while the impurity on the O site has host Zn atoms at sites 6,7, and 8, respectively. (**c**) The second nearest-neighbor complex centers ($C_{s}$/$C_{2v}$ symmetry) in the zinc-blend-type ZnO semiconductors. The complex can be formed by one impurity occupying the Zn site at 1 and the other at site 6. If the two impurities are identical, the symmetry is $C_{2v}$; otherwise, $C_{s}$ (see text).

In any defect calculations, the most important issue has been to give an adequate representation of the impurity perturbation $\overset{⃡}{P}$. To study the lattice dynamical behavior of defects using the GF method, we have appropriately constructed $\overset{⃡}{P}(\omega )$ by considering the (see Figure 2) effects of lattice relaxation to account for the impurity–host interactions. Lattice relaxation in the vicinity of substitutional impurities is estimated using Harrison’s semiempirical bond-orbital model [93]. In terms of Hartree–Fock atomic term values, this method provides simple analytical expressions for the change in impurity–host and host–host bond energies and suggests a computationally efficient and reasonably accurate way of estimating bond-length distortions. In the framework of RIM, the perturbation matrices $\overset{⃡}{P}(\omega )$ are constructed following the method described in Refs. [85,86,87]. To obtain $\overset{⃡}{P}\left(\omega \right),$ we have used the scaling properties of lattice relaxation caused by different isolated defects along with the trends of short-range interactions in different II–VI and III–V host crystals.

(a) Isolated Defects: $T_{d}$ Symmetry

In ZnO, the simplest defect responsible for impurity vibrational modes is an isolated substitutional impurity of $T_{d}$ symmetry (see Figure 2a): where the host lattice atom Zn (κ = 1) or O (κ = 2) [85,86,87] is replaced by an isoelectronic ${Be}_{Zn}\left(i\right)$, acceptor ${Li}_{Zn}\left(a^{-}\right),$ and donor $B_{Zn}\left(d^{+}\right)$ or $N_{O}\left(a^{-}\right)$ acceptor, respectively. In the framework of RIM, the perturbation matrix $\overset{⃡}{P}(\omega )$ includes both the changes in atomic masses at impurity sites and NN force constants (cf. Section 3). These changes are expressed by the following parameters:

(10a) $$\varepsilon_{1}=(M_{1}-M_{1}^{imp})/M_{1},$$

(10b) $$t=(A-A^{\prime })/A=(B-B^{\prime })/B=1\;–\;a,$$

or

(10c) $$\varepsilon_{2}=(M_{2}-M_{2}^{imp})/M_{2},$$

(10d) $$u=(A-A^{\prime \prime })/A=(B-B^{\prime \prime })/B=1\;–\;b,$$

for the impurity of mass $M_{1}^{imp}$ or $M_{2}^{imp}$ occupying either the site κ = 1 or 2, respectively. Following Vandevyver and Plumelle [86], we have considered the impurity–host interaction by a single dimensionless parameter t or u. The stipulation of a A = a B in Equations (10b) and (10d) for delineating the $\overset{⃡}{P}(\omega )$ matrix hardly affects the high-frequency LVMs. However, imposing this condition on $\overset{⃡}{P}(\omega )$ satisfies the rotational invariance requirement, which is explicitly invariant with respect to the translations and crystal-symmetry operations [85,86,87].

The constructions of a 15 × 15 full-size ${\overset{⃡}{G}}^{o}(\omega )$ and $\overset{⃡}{P}(\omega )$ matrices are reported in Ref. [86] for an isolated defect in the zb semiconductors. Considering the $T_{d}$ symmetry, we have decomposed ${\overset{⃡}{G}}^{o}(\omega )$ and $\overset{⃡}{P}(\omega )$ into blocks corresponding to the irreducible representations of the group [86]:

(11) $${\Gamma_{T}}_{d}=A_{1}⊗\;E\;⊗\;F_{1}⊗\;3F_{2}.$$

For isolated defects, the frequencies of local, gap, and in-band modes can be obtained in different irreducible representations by solving the real part of the determinant [86]:

(12) $$\prod_{\mu \Gamma }det|[\overset{⃡}{I}-{\overset{⃡}{G}}_{\mu \Gamma }^{o}(\omega )\;{\overset{⃡}{P}}_{\mu \Gamma }\left(\omega \right)]|\;=\;0.$$

Here, the ${\overset{⃡}{G}}_{\mu \Gamma }^{o}$ (ω) of the perfect lattice GF is projected onto the defect space, and ${\overset{⃡}{P}}_{\mu \Gamma }\left(\omega \right)$ is the perturbation matrix in each ($A_{1}$, E, $F_{1}$, and $F_{2}$) irreducible representation. One must note that the impurity modes in the $A_{1}$, E, and $F_{2}$ representations are Raman active, while the triply degenerate $F_{2}$ mode is IR, and is Raman active [85,86,87].

(b) NN Pair Defects: $C_{3v}$ Symmetry

The perturbation matrix $\overset{⃡}{P}(\omega )$ for a NN pair defect in zb ZnO involves two impurity atoms occupying the sites 1 and 2 (cf. Figure 2b), respectively, causing changes in the masses at impurity sites, i.e., $\varepsilon_{1}$ = ($M_{1}$ − $M_{1}^{imp}$)/$M_{1}$, $\varepsilon_{2}$ = ($M_{2}$ − $M_{2}^{imp}$)/$M_{2}$ and force constants between impurity–host atoms, i.e., t (1–2, 1–3, 1–4, 1–5) and u (2–1, 2–6, 2–7, 2–8). An effective force constant between impurities $F_{12}$ (≡ 1 − ab + $\Gamma_{12}$ = u + t − ut + $\Gamma_{12}$) is included (see Refs. [85,86,87] using $\Gamma_{12}$) to account for the changes in u, t of the isolated impurities involved in the formation of a pair defect. The term $F_{12}$ < 0 (or > 0) signifies stiffening (or softening) between the pair-bond. The pair defect of point group symmetry $C_{3v}$ involves eight atoms, which causes the size of the impurity space to increase to 24 × 24. The total representation of $C_{3v}$ in the 24-dimensional space group reported by Ludwig [94] is used to block-diagonalize the ${\overset{⃡}{G}}^{o}(\omega )$ and $\overset{⃡}{P}(\omega )$ matrices with each block along the diagonal belonging to the following irreducible representations:

(13) $${\Gamma_{C}}_{3v}={6A}_{1}⊗\;{2A}_{2}⊗8E.$$

From group-theoretical analysis, it is perceived that in the $A_{2}$ representation, the impurity atoms in the pair defect remain stationary. Thus, only the $A_{1}$ and E-type modes are optically active. As the degeneracies of the $F_{2}$ mode are lifted at each defect site, one expects to observe four LVMs for a pair defect with very light impurity atoms: two nondegenerate modes due to the movement of impurity atoms along the bond [i.e., $\omega_{1}$ ($A_{1}^{+}$ ← →) and $\omega_{4}$ ($A_{1}^{-}$ → →)] and two doubly degenerate modes as a result of their vibration perpendicular to it [i.e., $\omega_{2}$ ($E^{+}$↑↓) and $\omega_{3}$ ($E^{-}$↑↑)], generally with $\omega_{1}$ > $\omega_{2}$ > $\omega_{3}$ > $\omega_{4}$ (cf. Section 3.4). On the other hand, only two ($A_{1}$, E) impurity modes will appear for a pair defect involving a vacancy (or heavy) and a light impurity atom. We will use this perturbation model to account for the experimental results on impurity modes of NN pair defects (e.g., ${Al}_{Zn}-N_{O}$; ${Al}_{Zn}-P_{O}$) and for analyzing atypical Raman scattering spectroscopy data on impurity modes for complex centers involving Al donors and intrinsic defects.

(c) Complex Defects: $C_{s}$ or $C_{2v}$ Symmetry

The method used for the NN pair defect can be extended to define a perturbation matrix $\overset{⃡}{P}\left(\omega \right)$ for a complex center comprising three substitutional impurities (see Figure 2c) occupying sites 1 (cation), 2 (anion), and 6 (cation), respectively. Following the NN $C_{3v}$ pair defect case, we have considered the mass change parameter at the impurity site 6 in terms of $\varepsilon_{6}$ = ($M_{1}$−$M_{6}^{imp}$)/$M_{1}$ and the force constant variation between 6–2, 6–9, 6–10, and 6–11 impurity–host bonds by v = (A − $A^{\prime \prime \prime }$)/A = (B − $B^{\prime \prime \prime }$)/B = 1 − c. Similar to the NN pair defect, an effective force constant between the impurity atoms 2–6 (≡$F_{26}$) is also included. The point group symmetry for such a complex defect center is $C_{2v}$ if $\varepsilon_{1}$ = $\varepsilon_{6}$, otherwise $C_{s}.$ The NNN complex causes the size of the defect space to increase to 33 × 33. By constructing a total representation of $C_{2v}$/$C_{s}$ in the 33-dimensional space, we have block-diagonalized the ${\overset{⃡}{G}}^{o}(\omega )$ and $\overset{⃡}{P}(\omega )$ matrices belonging to the following irreducible representations [85,86,87]:

(14a) $${\Gamma_{C}}_{2v}={10A}_{1}⊗{6A}_{2}⊗\;{8B}_{1}⊗\;{9B}_{2},$$

and

(14b) $${\Gamma_{C}}_{s}={19A}_{1}⊗{14A}_{2}.$$

with the $A_{1}$, $B_{1}$, and $B_{2}$ ($A_{1}$ and $A_{2}$) types of vibrations being optically active. This perturbation model will be used to account for the experimental results on the impurity modes of NNN pair defects (e.g., ${Al}_{Zn}-O-{Li}_{Zn}$; $N_{O}-Zn-V_{O}$) and for analyzing the atypical Raman scattering spectroscopy data on the impurity modes of complex centers involving Al donors and intrinsic defects (see Section 3.3) in Al-doped ZnO.

## 3. Numerical Computation Results and Discussion

### 3.1. Theoretical Framework of Phonons

Significant interest has recently grown in ZnO due to its potential use in developing devices for transport electronics and UV optoelectronics. Lattice dynamics plays an important role in comprehending electron transport and the interaction of phonons with charge carriers. In polar semiconductors, when free charges are excited high in the conduction band, they relax to the ground states via the Fröhlich interaction involving LO phonons. One, therefore, expects the dynamics of phonon population to strongly impact the performance of high-speed electronic devices. Despite extensive measurements known for assessing the structural and electrical properties of doped wz materials, there are limited spectroscopic studies available on zb ZnO for appraising their phonon, bonding, and impurity vibrational characteristics [95].

**Figure 3:**
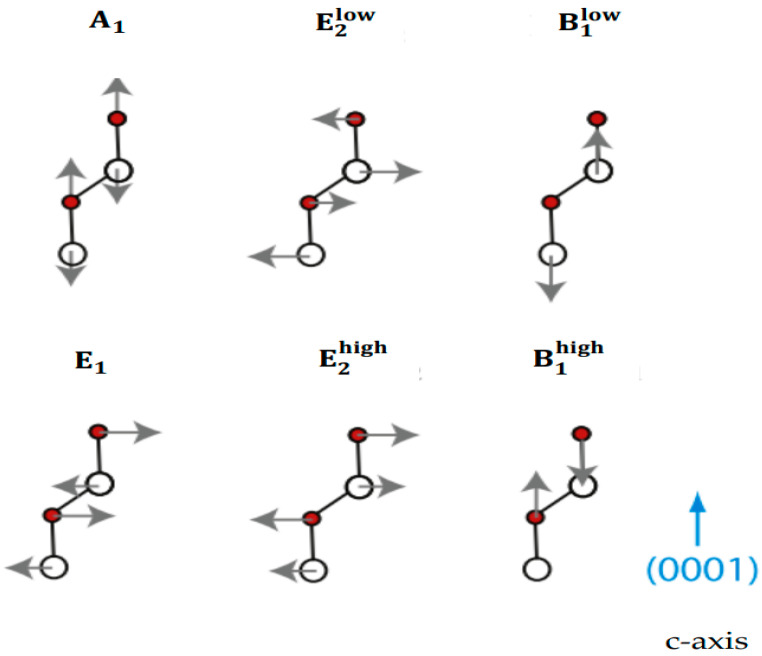
The atomic displacements of optical phonon modes in the wz ZnO crystal structure. The white-colored spheres represent the Zn atoms, while the red spheres indicate the O atoms.

As the cohesive energy of ZnO is very close to that of the zb material, it can be grown either in the wz and/or zb phases. The wz ZnO with 4 atoms/unit cell and space group $C_{6v}^{4}$ is a non-centrosymmetric, having 12 normal modes as compared to 6 modes in the zb ($T_{d}^{5}$) lattice with 2 atoms/unit cell. Based on group theory, the total representation of zone center optical phonons can be classified as [66]:

(15) $$\Gamma_{opt}={2A}_{1}+2B_{1}+2E_{1}^{2}+2E_{2}^{2}$$

where the superscript 2 on $E_{1}$ and $E_{2}$ indicates double degeneracy. Contrary to zb, the normal modes of the wz phase are linked to different symmetry representations. Among the 12 modes, one set of $A_{1}$ and $E_{1}^{2}$ are acoustic, and the other nine phonons are optical. At Γ point, the acoustic modes vanish, while one $A_{1}$, two $B_{1}$, one $E_{1}$, and two $E_{2}$ optical phonons are non-vanishing. The polar $A_{1}$ and $E_{1}$ modes are Raman and IR active, while the nonpolar $E_{2}$ phonons are only Raman active. The $E_{2}$ and $E_{1}$ modes exhibit in-plane atomic vibrations, whereas the atoms in $A_{1}$ modes vibrate along the c-axis (see Figure 3).

#### Lattice Dynamics of ZnO

**Figure 4:**
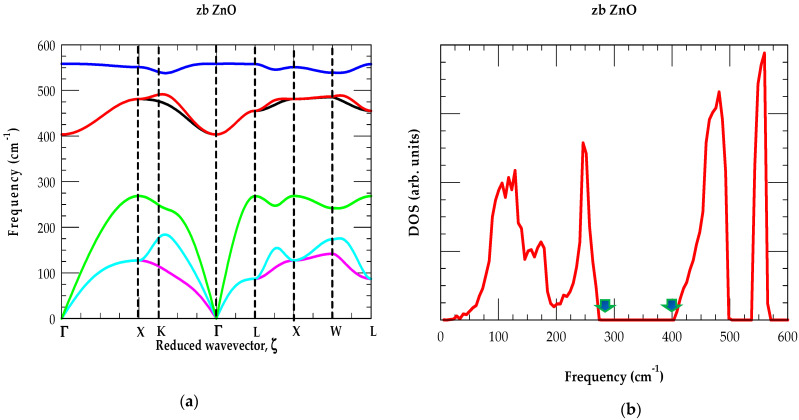
(**a**) Rigid-ion model calculations of the phonon dispersions along high-symmetry directions for the zb ZnO material; (**b**) rigid-ion model calculations of the one-phonon density of states exhibiting the phonon gap in zb ZnO occurring between the maximum acoustic modes and the minimum optical phonons region (shown by green-colored vertical arrows) between ~275–405 cm^−1^.

The phonon dispersions $\omega_{j}(\vec{q})$ of the wz ZnO material are measured using INS and calculated by the first principles method [65]. At a few critical points, the phonon modes are also achieved by IR reflectivity/absorption and Raman scattering spectroscopy. In zb ZnO, the phonon dispersions are displayed in Figure 4a by RIM along high-symmetry (Γ→X→ K→ Γ→ L→ X→ W→ L) directions in the BZ. The theoretical results have agreed reasonably well with the RSS [65] data. A simulated one-phonon DOS $g\left(\omega \right)$ is shown in Figure 4b.

Inspection of Figure 4a,b confirms that the optical and acoustic phonons of zb ZnO are affected by the light O (16.0 amu) and heavier Zn (65.38 amu) atomic masses, respectively. The first and second peaks in the low-frequency region of $g\left(\omega \right)$ are associated (see Figure 4b) with the average values of the $\omega_{TA}$ and $\omega_{LA}$ modes, while two high-frequency peaks correspond to the average $\omega_{TO}$ and $\omega_{LO}$ phonons, respectively. In $g\left(\omega \right),$ the phonon gap (~275–405 cm^−1^) between the maximum acoustic and minimum optical phonons is caused by the large mass difference between the O and Zn atoms. In Table 1, we have listed the main phonon frequency features at high critical points for both the wz and zb ZnO materials.

### 3.2. Chemical Trends of Impurity–Host Interactions

The vibrational properties of defects in wz ZnO are studied by Raman scattering spectroscopy for the closest mass ${Li}_{Zn}\left(a^{-}\right)acceptor,{Be}_{Zn}\left(i\right)$ isoelectronic, and $B_{Zn}\left(d^{+}\right)$ donors. Similar measurements also exist for sodium ${Na}_{Zn}\left(a^{-}\right)$, magnesium ${Mg}_{Zn}\left(i\right),$ and aluminum ${Al}_{Zn}\left(d^{+}\right)$ dopants [24,25,26,27,28,29,30,31,32,33,34,35,36]. A careful analysis of the LVMs for charged defects in zb crystals has conveyed a very important revelation. The estimated changes in the NN force constants are ascribed to the effects of the electric field produced by charged impurities [85,86,87]. This effect instigates the redistribution of charge around an isolated impurity to produce changes in the covalency (or ionicity) between the impurity–host bond. In the II–VI materials, a simple empirical relationship provides corrections to ∆t in the NN force constants of the closest mass isoelectronic Mg (i) and donor Al ($d^{+}$) or isoelectronic Be (i) and acceptor Li ($a^{-}$), occupying the cation sites and exhibiting the following trends [85,86,87]:

(16a) $$∆t\left\{d_{II}^{+}-i_{II}\right\}\;<\;\mathrm{stiffening},$$

(16b) $$∆t\left\{a_{II}^{-}-i_{II}\right\}\;>\;\mathrm{softening}.$$

Similarly, the values of ∆u between the closest mass isoelectronic S (i) and an acceptor P ($a^{-}$) occupying the anion site reveals:

(16c) $$∆u\left\{a_{VI}^{-}-i_{VI}\right\}\;<\;\mathrm{stiffening}.$$

and we expect

(16d) $$∆u\left\{d_{VI}^{+}-i_{VI}\right\}\;>\;\mathrm{softening},$$

for an isoelectronic S (i) and a donor Cl ($d^{+}$) that occupies the anion sites, respectively. Absolute values of relative variations in ∆t and ∆u for the single charged ($a^{-}$, $d^{+}$) and isoelectronic (i) defects producing LVMs in the II–VI and III–V compounds are seen within 30%. Although these correlations are found independent of the long-range Coulomb forces, we have strongly argued that the charged impurities in semiconductors affect only the short-range forces via the redistribution of electron-charge density. These arguments are supported in a self-consistent super-cell study by Baraff et al. [76], where the electronic-charge density contours are simulated for both the perfect GaP and imperfect Ga*P*:O systems to mark the evidence of weak bonding between the $O_{P}$–Ga bonds.

We believe that these unique and valuable trends in force variations (Equations (16a) and (16d)) are significant to serve as a good starting point to make predictions of impurity modes for both the isolated and complex defect centers in zb ZnO. Next, we will start to verify these arguments in learning the composition x, that is dependent on the long-wavelength optical phonons in ${Be}_{x}{Zn}_{1-x}O$ alloys. The results of the impurity modes reported in Section 3.3 using the GF simulations for isolated and complex defect centers in zb ZnO will be compared/contrasted against the existing Raman scattering and FTIR data [24,25,26,27,28,29,30,31,32,33,34,35,36].

#### Long-Wavelength Optical Phonons in ${Be}_{x}{Zn}_{1-x}O$ Alloys

To understand the composition-dependent long-wavelength optical phonons in ternary ${Be}_{x}{Zn}_{1-x}O$ alloys, we have used the MREI approach [89,90]. This model assumes that the x portion of the NN of O has Be atoms with 1 − x sharing the Zn atoms. The NN of Be and Zn has O atoms. A long-range Coulomb interaction is included as a local field. The necessary parameters (cf. Table 2) of the constituting bonds (Be-O; Zn-O; Be-Zn) are obtained using the atomic masses and effective charges by relating them to $\omega_{TO}$, $\omega_{LO}$ phonons and LVM as well as in-band/gap mode frequencies.

The LVM and in-band/gap mode obtained by the GF method at the extreme composition limits x → 0 for isolated ${Be}_{Zn}$ (in zb ZnO) and x → 1 ${Zn}_{Be}\;($in BeO) defects are used for evaluating the necessary MREI parameters:

(17a) $$\omega_{TO,AB}^{2}=\frac{F_{AB,0}}{\mu_{AB}},$$

(17b) $$\omega_{TO,AC}^{2}=\frac{F_{AC,0}}{\mu_{AC}}(1-\theta ),$$

(17c) $$\omega_{in-band/gap}^{2}=\frac{F_{AC,0}+F_{BC,0}}{m_{C}},$$

and

(17d) $$\omega_{LVM}^{2}=\frac{F_{AC,0}+F_{BC,0}}{m_{B}}(1-\theta ).$$

**Figure 5:**
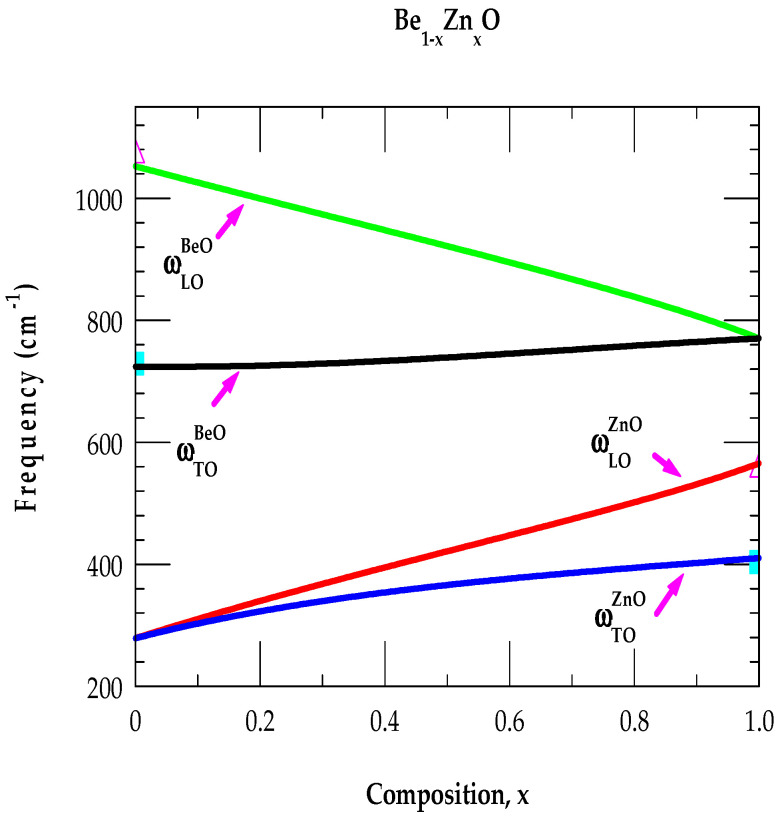
Simulated two-phonon-mode behavior of ${Be}_{1-x}{Zn}_{x}O$ alloys based on a modified random iso-displacement model.

The model [90] offers a simple criterion for predicting the two-phonon mode behavior for $AB_{x}C_{1-x}$ alloys. If $m_{A}$, $m_{B}$,$\;and\;m_{C}$ are the masses of the A, B, and C atoms, such that ($m_{B}$ < $m_{C})$ has the reduced mass $\mu_{AC}$ of A and C having $m_{B}\;$< $\mu_{AC}$, then, the $AB_{x}C_{1-x}\;$ alloy displays two-phonon-mode behavior. For A = O, B = Be, and C = Zn, the above criteria are valid for ${Be}_{x}{Zn}_{1-x}O$. Calculations of x-dependent optical phonons, which are displayed in Figure 5, with the parameter values listed in Table 2i,ii, have confirmed the two-phonon-mode behavior. Our results are in very good agreement with a recent study reported for wz ${Be}_{x}{Zn}_{1-x}O$ alloys [96].

### 3.3. Vibrational Modes of Isolated Defects in zb ZnO

For ${Be}_{x}{Zn}_{1-x}O,$ the GF methodology (cf. Section 3.2) is used to calculate the impurity vibrational modes of the isoelectronic ${Be}_{Zn}\left(i\right)$ and ${Zn}_{Be}(i)$ defects in the extreme composition limits: x → 0 (ZnO:Be) and x → 1 (BeO:Zn). Careful inclusion of the appropriate perturbations for lighter ${Be}_{Zn}$ in zb ZnO has predicted a high-frequency LVM at ~780 cm^−1^ and an in-band impurity mode at a lower frequency of ~280 cm^−1^ for heavier ${Zn}_{Be}$ in zb BeO.

#### Impurity Modes of Closest Mass Isoelectronic Donor and Acceptor on the Zn (O) Sites in ZnO

**Figure 6:**
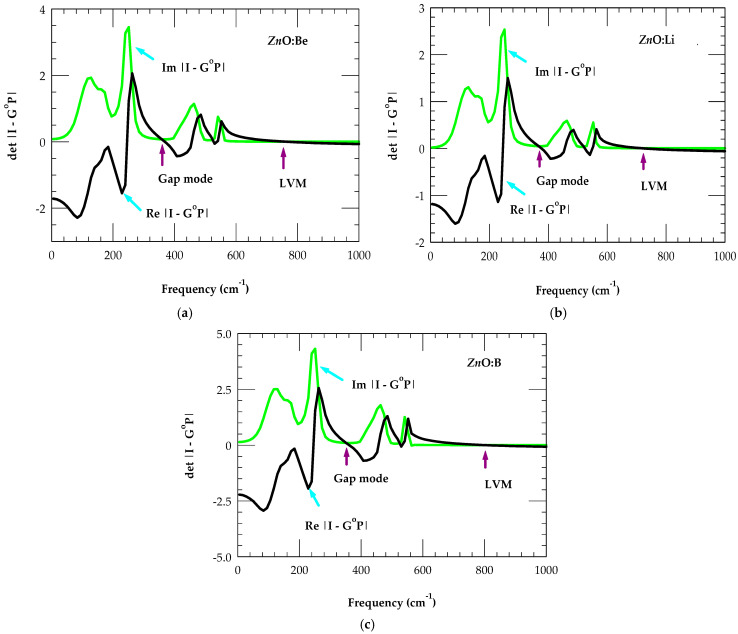
The calculated real (black line) and imaginary (green line) parts of the det| I − G^o^P|(cf. Section 2.3.3) in the $F_{2}$ representation. Crossing zero of the real part of det |I – G^o^P| provides local and gap modes (see Table 3) for (**a**) ${Be}_{Zn},(b)$ ${Li}_{Zn}$, and (**c**) $B_{Zn}\;inzb$ ZnO (see text).

Calculations of the LVM for ${Be}_{Zn}\;(i)$ has helped us to estimate the impurity vibrational modes of the closest mass lithium acceptor ${Li}_{Zn}$ ($a^{-}$) and boron donor $B_{Zn}\;(d^{+})$ in zb ZnO. Moreover, considering the mass change at impurity sites, the GF simulations are performed by including appropriate force variations using Equations (16a) and (16b). In Table 3, we have listed the predicted values of LVMs and GMs for several isolated impurity atoms occupying the Zn (O) site in the zb ZnO. The impurity vibrational modes are estimated from the results of the real and imaginary parts of det |I-$G^{o}P|$, displayed as a function of frequency. We have used Equation (12) and Figure 6a,c for assessing LVMs and GMs when the real part of det |I-$G^{o}P|$ crosses to zero.

The results reported in Table 3 and Figure 6a,c have revealed interesting characteristics. Although the mass of Li (B) is lower (higher) than that of the isoelectronic Be, the GF calculations predicted a LVM at a lower (higher) ~740 cm^−1^ (~804 cm^−1^) frequency for ${Li}_{Zn}(a^{-})$ ($B_{Zn}\;(d^{+}))$. The shifts in impurity modes are linked to NN force constant variations, causing softening (stiffening) between the ${Li}_{Zn}-O$ ($B_{Zn}-O)$ bonds. While no measurements of impurity modes exist for these defects in the zb ZnO, our results have agreed very well with the Raman scattering (FTIR) studies of Li (B)-doped [27,28] wz ZnO nanocrystals for the Li-O (B-O) mode in the LiO_2_ (BO_2_) configuration. Aside from the LVMs, each light impurity in zb ZnO predicts the GM, with its frequency decreasing with the increase in mass from ${Li}_{Zn}$ → ${Be}_{Zn}$ → $B_{Zn}$. These results have provided strong corroborations to an earlier study by Vandevyver and Plumelle [86], where the authors have predicted both the LVMs and GMs for light impurities occupying the Zn sites in zb ZnS.

**Figure 7:**
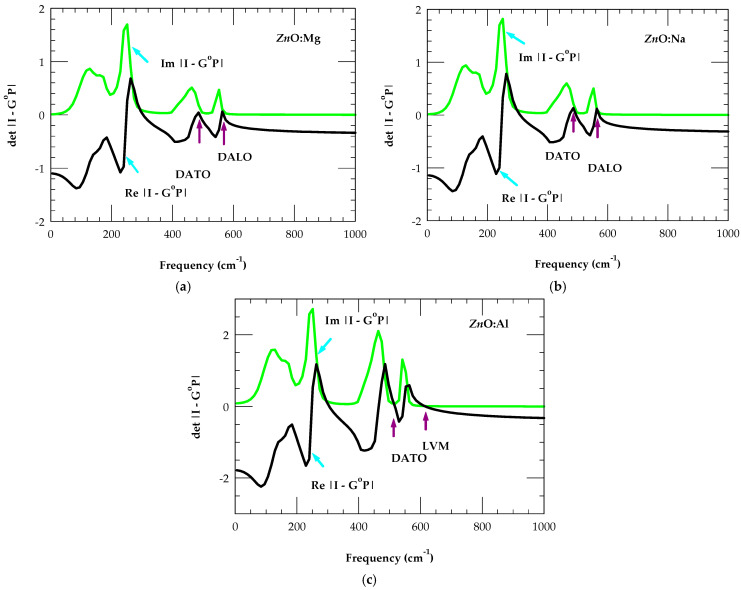
The calculated real (black line) and imaginary (green line) parts of the det | I − G^o^P | (cf. Section 2.3.3) in the $F_{2}$ representation showing local, gap, and/or defect-activated (DATO, DALO) modes (see Table 3) of (**a**) ${Mg}_{Zn},(b)$ ${Na}_{Zn},$ and (**c**) ${Al}_{Zn}\;inzb$ ZnO (see text).

Similar calculations of the impurity modes are displayed in Figure 7a,c for the closest mass Mg isoelectronic ${Mg}_{Zn}\left(i\right)$, Na acceptor $\;{Na}_{Zn}$ ($a^{-}$), and Al donor ${Al}_{Zn}(d^{+})$ in zb ZnO.

With respect to ${Mg}_{Zn}\left(i\right),$ the changes in ∆t for ${Na}_{Zn}$ ($a^{-}$) and ${Al}_{Zn}(d^{+})$ have indicated softening (stiffening) between the ${Na}_{Zn}$-O (${Al}_{Zn}$-O) bonds. Thus, only the ${Al}_{Zn}(d^{+})$ donor exhibited the LVM while all impurities (${Na}_{Zn}$, ${Mg}_{Zn},and{Al}_{Zn}$) indicated the possibilities of GMs. Consistent with the closest masses of the Li, Be, and B dopants, the frequencies of GMs are shown to decrease with the increase in impurity mass ${Na}_{Zn}\to {Mg}_{Zn}\to {Al}_{Zn}$. In the absence of experimental results for zb ZnO, our theoretical simulations of defect-activated optical phonons (see Table 3) are compared reasonably well with the limited Raman scattering and/or FTIR data in wz ZnO. In some reports [24,25,26,27,28,29,30,31,32,33,34,35,36], many researchers have quoted the impurity-activated optical phonons for ${Mg}_{Zn}$ and other impurities (Mn, Co, and Fe) as LVMs. Such assignments are inaccurate and need to be corrected.

### 3.4. Impurity Vibrational Modes of Complexes in ZnO

Similar to the II–VI semiconductors, double-doping with group III (Y = B, Al, Ga, and In) $Y_{Zn}$ ($d^{+}$) atoms and group V (X = N, P, As, and Sb) $X_{O}\left(a^{-}\right)$ impurities in ZnO can create NN “donor-acceptor” $Y_{Zn}(d^{+})$-$X_{O}(a^{-})$ pairs of $C_{3v}$ symmetry. A simultaneous doping of Li with group III defects can also form second NN $Y_{Zn}(d^{+})$-O-${Li}_{Zn}(a^{-})$ complex centers of $C_{s}$ symmetry. In Table 4, we have reported the results of our GF calculations for the LVMs of different impurity complex centers. In zb ZnO, the calculations of NN ($C_{3v}$) and second NN ($C_{s}$) pairs are performed by keeping the same values of force constants that are estimated for the isolated defects. Interesting trends that were perceived in the simulations of the LVMs are worth recognizing: (a) The NN ${Al}_{Zn}(d^{+})$-$X_{O}(a^{-})$ pairs of $C_{3v}$ symmetry reveals that only two LVMs are linked to the vibration of ${Al}_{Zn}\left(d^{+}\right)$—the other two modes associated with $X_{O}(a^{-})$ fall, however, into the optical phonon regions. The average frequency of the ${Al}_{Zn}$ LVM $\bar{\omega }$ stays nearly constant when heavier $X_{O}(a^{-})$ impurities are involved in the pair. (b) The second NN $Y_{Zn}(d^{+})$-$O-{Li}_{Zn}(a^{-})$ pairs of $C_{s}$ symmetry were confirmed to cause six vibrational modes, three each of the light $B_{Zn}$ (${Al}_{Zn})$ donor and isotopic ${Li}_{Zn}$ acceptors, respectively. The average frequency of the splitting of the LVM for each isolated defect in the complex center falls well within their triply degenerate values.

These outcomes have provided valuable evidence for the site selectivity of different light substitutional defects from groups I and III, as well as their atomic interactions with the host lattice atoms in zb ZnO. This information will be quite effective for assessing the evidence of the microstructural configuration of NN and second NN complexes, particularly the creation of impurity pairs involving double dopants. Similar to many II–VI semiconductors, the vibrational study by the GF method has provided support for the association of different impurities with intrinsic ($V_{Zn}$; $V_{O}$) defects. We feel that the LVMs of different impurity complexes in zb ZnO will encourage spectroscopists to use FTIR and/or Raman scattering spectroscopy to check our theoretical conjectures.

## 4. Concluding Remarks

Many scientists and engineers working in nanoelectronics/photonics have focused, in recent years, on discovering novel materials for designing devices to satisfy the growing needs of the high-T electronics, healthcare, photovoltaic, and automotive industries. Devices based on WBG GaN, SiC, and ZnO $(E_{g}$~3.37 eV) [97,98,99,100] can be used to cover nearly the same wavelengths. However, the ZnO material of exciton-binding energy (~60 meV) has been preferred for the fabrication of laser diodes with a lower threshold for excitons for operation at a high efficiency and temperature. Despite many valuable characteristics of ZnO, an important issue has arisen in achieving reproducible p-type conductive layers. Doping of ZnO by monovalent lithium can be a prospective approach to compensate n-type conductivity by intrinsic defects and intentionally doped trivalent (B, Al) atoms [17]. In ZnO: Al (Li), although SEM and PL measurements [65,66,67] are used for determining the morphology and investigating their optical properties, only limited impurity vibrational features exist when using FTIR and Raman spectroscopy [24,25,26,27,28,29,30,31,32,33,34,35,36,37,38]. In the framework of a realistic RIM [88] and by integrating accurate perturbation models into the Green’s function method, we have meticulously simulated the impurity vibrational modes of ${Al}_{Zn}$ ${(Li}_{Zn})$ and their bonding to form NN ${[Al}_{Zn}{-N}_{O}$] pairs and second NN ${[Al}_{Zn}{-O-Li}_{Zn};{V_{Zn}-O{-Li}_{Zn}]}_{\kappa }$ complexes. We strongly feel that these phonon characteristics in doping ZnO material will encourage spectroscopists to perform similar measurements to confirm our theoretical conjectures.

## Figures and Tables

**Table 1:** Comparison of the major phonon frequencies in ${cm}^{-1}$ for the wz and zb ZnOs [65,66].

wz ZnO	$\omega \;{cm}^{-1}$	zb ZnO	$\omega \;{cm}^{-1}$
$A_{1}^{LO}$	560 (584, 575)	$\omega_{LO(\Gamma )}$	558
$E_{1}^{LO}$	556 (595, 580, 588)	$\omega_{TO(\Gamma )}$	403
$B_{1}^{high}$	552	$\omega_{LO(X)}$	551
$E_{2}^{high}$	440 (438, 439)	$\omega_{TO(X)}$	487
$E_{1}^{TO}$	409 (410, 411)	$\omega_{LO(L)}$	561
$A_{1}^{TO}$	391 (380, 379)	$\omega_{LO(L)}$	443
$B_{1}^{low}$	261	$\omega_{TA(X)}$	128
$E_{2}^{low}$	91 (100)	$\omega_{TA(L)}$	93

Refs. [65,66]

**Table 2:** (**i**) Parameters required for optimizing the MREI model parameters [90] for calculating (**ii**) the two-mode behavior in $O{Be}_{x}{Zn}_{1-x}$ ternary alloys.

**(i)**	$\omega_{TO}\;({cm}^{-1})$	$\omega_{LO}\;({cm}^{-1})$	$\omega_{l}$ $\left({cm}^{-1}\right)$	$\varepsilon_{\infty }$	$a_{o}\;(Å)$
**BeO**	728	1073	BeO:Zn = 280	3.10	3.81
**ZnO**	403	558	ZnO:Be = 775	5.32	4.504
$\left(ii\right){AB_{x}C_{1-x}}^{\left(a\right)}O{Be}_{x}{Zn}_{1-x}$					
$e_{AB}^{*}$			3.10		
$e_{AC}^{*}$			5.32		
$F_{AB0}$			2.99E+5 dyn/cm		
$F_{AC0}$			2.40E+5 dyn/cm		
$F_{BC0}$			5.96E+4 dyn/cm		
**θ**			0.12		

^(a)^ Ref. [90]

**Table 3:** Comparison of GF calculations [of gap (GMs), local vibrational (LVMs), and in-band modes] with experimental data of the closest mass isoelectronic, acceptor, and donor defects in ZnO.

System	Impurity Modes ^(a)^	Impurity Modes (Others) ^(b)^	Δf/f
ZnO:Li	372 cm^−1^ (GM), 732 cm^−1^ (LVM)	735 cm^−1^	−0.17
ZnO:Be	363 cm^−1^ (GM), 775 cm^−1^ (LVM)	~760, ~775 cm^−1^	−0.50
ZnO:B	354 cm^−1^ (GM), 804 cm^−1^ (LVM)	806, 819, 880 cm^−1^	−0.82
ZnO:Na	305 cm^−1^ (GM), 486, 541 cm^−1^	270, 513 cm^−1^	0.07
ZnO:Mg	298 cm^−1^ (GM), 467, 551, 574 cm^−1^	250, 304, 490, 562, 568 cm^−1^	−0.25
ZnO:Al	295 cm^−1^ (GM), 497, 545, 612 (LVM) cm^−1^	475, 500, 560, 581, 623 cm^−1^	−0.55
ZnO:N	497, 575 cm^−1^	277, 497, 512, 552, 575, 582 cm^−1^	−0.1
ZnO:P	372 cm^−1^ (GM), 466, 543 cm^−1^	284, 361, 486 cm^−1^	−0.14
ZnO:As	273, 470, 545 cm^−1^	220, 266, 550 cm^−1^	−0.17
ZnO:Sb	235, 470, 545 cm^−1^	235, 534 cm^−1^	−0.2

^(a)^ Ours; ^(b)^ refs. [24,25,26,27,28,29,30,31,32,33,34,35,36]

**Table 4:** In zb ZnO, our predictions of localized vibrational modes using the Green’s function method for various complex defect centers of $C_{3v}$ and C_s_ symmetries.

zb ZnO	Modes (cm^−1^)	Average Impurity Mode	∆f/f
$C_{3v}:{Al}_{Zn}-N_{O}$	$A_{1}=648$	$\bar{\omega }=$ 627	−0.55
	E=616		−0.10
$C_{3v}:{Al}_{Zn}-P_{O}$	E=616	$\bar{\omega }=616$	−0.55
	$A_{1}=602$		−0.08
$C_{3v}:{Al}_{Zn}-{As}_{O}$	E=616	$\bar{\omega }=611$	−0.55
	$A_{1}=602$		−0.06
$C_{3v}:{Al}_{Zn}-{Sb}_{O}$	E=616	$\bar{\omega }=616$	−0.55
	$A_{1}=602$		−0.04
$C_{s}$:$B_{Zn}-{6Li}_{Zn}$	$A_{2}=813$	$\bar{\omega }=808$	−0.83
	$A_{1}=811$		−0.17
	$A_{1}=799$		
	$A_{1}=775$	$\bar{\omega }=763$	
	$A_{2}=772$		
	$A_{1}=742$		
$C_{s}$:$B_{Zn}-{7Li}_{Zn}$	$A_{2}=808$	$\bar{\omega }=802$	−0.83
	$A_{1}=804$		−0.17
	$A_{1}=793$		
	$A_{1}=736$	$\bar{\omega }=727$	
	$A_{2}=734$		
	$A_{1}=711$		
$C_{s}$:${Al}_{Zn}-{6Li}_{Zn}$	$A_{2}=616$	$\bar{\omega }=612$	−0.55
	$A_{1}=613$		−0.17
	$A_{1}=606$		
	$A_{2}=782$	$\bar{\omega }=774$	
	$A_{1}=781$		
	$A_{1}=760$		
$C_{s}$:${Al}_{Zn}-{7Li}_{Zn}$	$A_{2}=616$	$\bar{\omega }=612$	−0.55
	$A_{1}=613$		−0.17
	$A_{1}=606$		
	$A_{2}=739$	$\bar{\omega }=732$	
	$A_{1}=738$		
	$A_{1}=718$		
$C_{s}$:${Ga}_{Zn}-{6Li}_{Zn}$	$A_{2}=782$	$\bar{\omega }=774$	−0.45
	$A_{1}=781$		−0.17
	$A_{1}=758$		
$C_{s}$:${Ga}_{Zn}-{7Li}_{Zn}$	$A_{2}=758$	$\bar{\omega }=737$	−0.45
	$A_{1}=737$		−0.17
	$A_{1}=717$		
$C_{s}$:${In}_{Zn}-{6Li}_{Zn}$	$A_{2}=782$	$\bar{\omega }=774$	−0.35
	$A_{1}=781$		−0.17
	$A_{1}=752$		
$C_{s}$:${In}_{Zn}-{7Li}_{Zn}$	$A_{2}=738$	$\bar{\omega }=730$	−0.35
	$A_{1}=737$		−0.17
	$A_{1}=716$		
$C_{s}$:$V_{Zn}-O-{Al}_{Zn}$	$A_{2}=616$	$\bar{\omega }=611$	1.00
	$A_{1}=613$		−0.55
	$A_{1}=603$		
$C_{s}$:$V_{Zn}-O-B_{Zn}$	$A_{2}=804$	$\bar{\omega }=797$	1.00
	$A_{1}=802$		−0.83
	$A_{1}=784$		
$C_{s}$:$V_{O}-Zn-N_{O}$	$A_{2}=574$	$\bar{\omega }=570$	1.00
	$A_{1}=573$		−0.1
	$A_{1}=562$		

## Data Availability

The data that support the findings of this study are available from the author upon reasonable request.
